# Artificial intelligence‐enabled innovations in cochlear implant technology: Advancing auditory prosthetics for hearing restoration

**DOI:** 10.1002/btm2.10752

**Published:** 2025-01-09

**Authors:** Guodao Zhang, Rui Chen, Hamzeh Ghorbani, Wanqing Li, Arsen Minasyan, Yideng Huang, Sen Lin, Minmin Shao

**Affiliations:** ^1^ Department of Otorhinolaryngology The Dingli Clinical College of Wenzhou Medical University, Wenzhou Central Hospital Wenzhou China; ^2^ Institute of Intelligent Media Computing Hangzhou Dianzi University Hangzhou China; ^3^ Shangyu Institute of Science and Engineering Co.Ltd. Hangzhou Dianzi University Shaoxing China; ^4^ Department of Otorhinolaryngology The Third Affiliated Hospital of Wenzhou Medical University Wenzhou China; ^5^ Faculty of General Medicine University of Traditional Medicine of Armenia (UTMA) Yerevan Armenia; ^6^ Department of Otolaryngology The First Affiliated Hospital of Wenzhou Medical University Zhejiang China

**Keywords:** adaptive signal processing, artificial intelligence, auditory rehabilitation, cochlear implants, speech perception

## Abstract

This comprehensive review explores the implications of artificial intelligence (AI) in addressing cochlear implant (CI) issues and revolutionizing the landscape of auditory prosthetics. It begins with an overview of ear anatomy and hearing loss, then explores a review of CI technology and its current challenges. The review emphasizes how advanced AI algorithms and data‐driven approaches enhance CI adaptability and functionality, enabling personalized rehabilitation strategies and improving speech enhancement. It highlights diverse AI applications in auditory rehabilitation, including real‐time adaptive control mechanisms and cognitive hearing assistants that help users manage their auditory health. By outlining innovative pathways and future directions for AI‐enhanced CIs, the paper sets the stage for a transformative shift in auditory prosthetics, aiming to improve the quality of life for individuals with hearing loss.

**Abbreviations**
ACE: advanced combination encoder
AI: artificial intelligence
CI: cochlear implant
CIS: continuous interleaved sampling
HiRes: high resolution
ML: machine learning
RF: radio frequency

## 1 INTRODUCTION

Hearing loss is a complex sensory impairment that significantly affects the quality of life, communication capabilities, and social interactions across all age groups. Untreated hearing loss can delay speech and language development in children, subsequently impacting academic performance and social integration. For adults, hearing loss can lead to challenges in the workplace, potential isolation, and an increased risk of mental health issues, such as depression and anxiety. The cumulative effects of hearing loss underscore the importance of early detection, appropriate intervention, and continuous support to enhance the overall well‐being and social inclusion of affected individuals.^1^, ^2^, ^3^

The etiology of hearing loss is diverse, encompassing genetic factors, environmental influences, medical conditions, and acquired causes.^4^ Among the various types of hearing loss, conductive hearing loss arises from issues in the outer or middle ear, such as ear infections, otosclerosis, or perforated eardrums. In cases where conventional hearing aids are insufficient for adequate auditory rehabilitation, cochlear implants (CIs) offer a transformative solution.^5^

CIs have revolutionized the management of severe to significant hearing loss by providing auditory perception to individuals who receive limited or no benefit from conventional hearing aids. The CI system consists of both external and internal components:

- External components

1. *Microphone*: Captures sound from the environment, including speech, and converts it into electrical signals.
2. *Speech processor*: This device receives electrical signals from the microphone, filters them, and processes them to extract relevant speech and environmental sounds.
3. *The Transmitter coil*: It sends processed signals to the internal components of the CI via radio frequency (RF) waves.

- Internal components

1. *Receiver/stimulator*: This device receives the processed signals from the transmitter coil and converts them into electrical impulses.
2. *Electrode array*: Directly stimulates the auditory nerve fibers, allowing the user to perceive sound.

**FIGURE 1:**
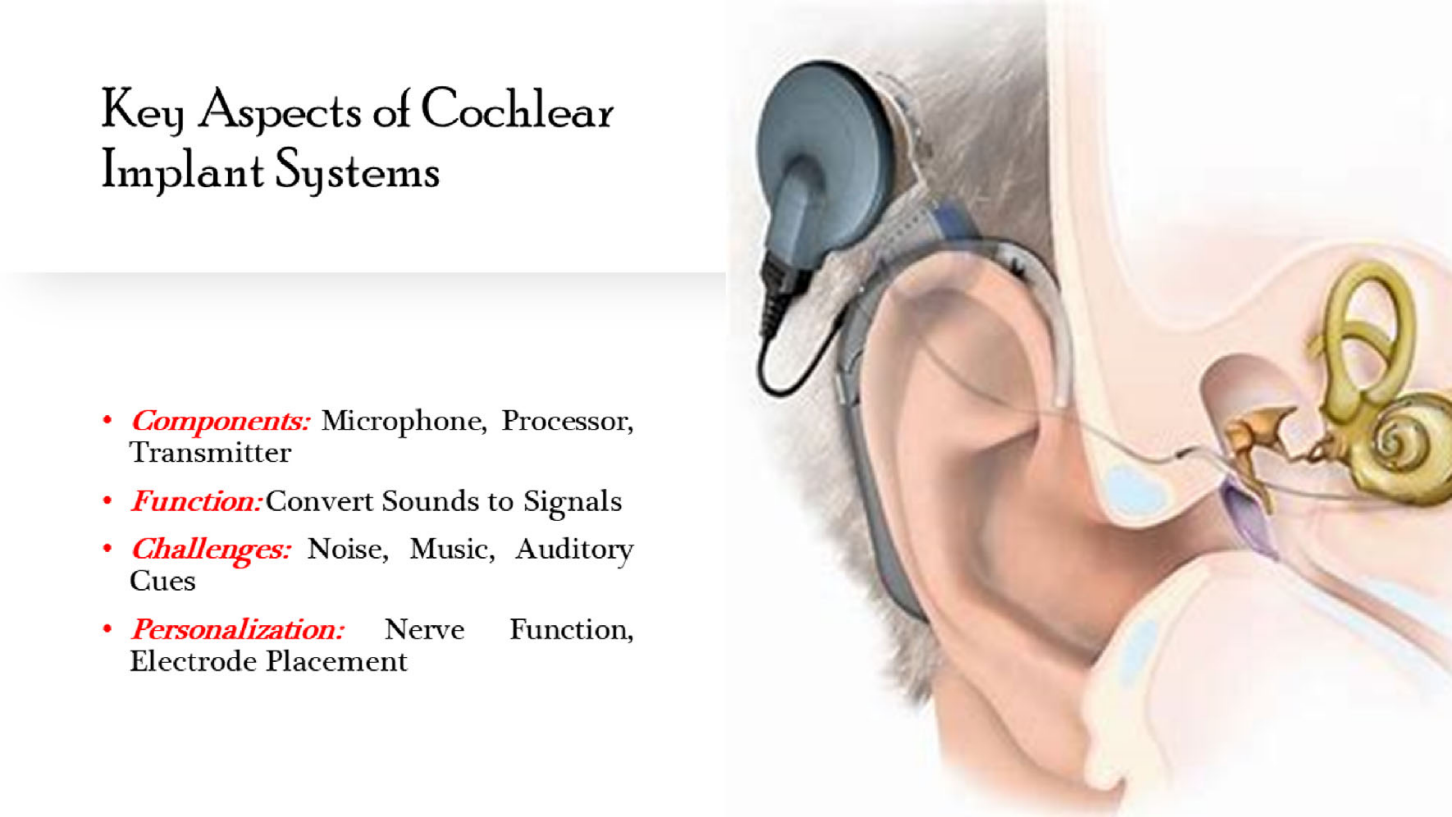
The key aspects of CI systems (the idea of the picture is captured from Lenarz^12^).

This setup allows external components to effectively gather and convert sound into electrical signals that can be used by an internal device to stimulate the auditory nerve and facilitate hearing in individuals with severe hearing loss (Figure 1). The electrical impulses generated by the receiver‐stimulator travel along the electrode array, directly stimulating the auditory nerve fibers.^3^, ^6^, ^7^

Despite remarkable advancements in CI technology, several challenges remain, including limitations in speech comprehension, particularly in noisy environments, and difficulties in perceiving music and subtle auditory nuances. Unlike previous studies that primarily focused on the mechanical and acoustic aspects of CIs, this review emphasizes the integration of advanced AI algorithms and data‐driven approaches to enhance auditory outcomes. It highlights innovative developments such as noise reduction techniques, adaptive signal processing, and cognitive computing capabilities that empower users to manage their auditory health more effectively. Furthermore, the paper discusses the potential for personalized and intelligent devices that can adapt to individual user needs and environments, thereby improving speech perception and overall quality of life for CI users. By synthesizing current research and projecting future trends, this work underscores the importance of interdisciplinary collaboration among researchers, clinicians, and engineers, setting the stage for future innovations that could redefine auditory rehabilitation.^8^, ^9^, ^10^, ^11^

Artificial intelligence (AI) techniques, such as machine learning (ML), offer opportunities to improve speech processing algorithms, personalize rehabilitation strategies, and optimize device settings based on individual user feedback. By leveraging AI‐driven innovations, researchers and clinicians aim to unlock the full potential of CI technology and ultimately improve outcomes. The role of AI in healthcare innovation has undergone significant expansion in recent years, revolutionizing various aspects of medical practice, research, and technological development. AI, encompassing advanced ML algorithms and deep‐learning techniques, has demonstrated remarkable potential for enhancing diagnostic accuracy, treatment efficacy, and healthcare delivery efficiency.^13^, ^14^ As healthcare systems face rising costs, limited access, and the need for personalized care, AI has emerged as a transformative solution.^15^ Evaluated AI's integration of AI in healthcare and examined its role in clinical decision‐making, hospital management, medical image analysis, and patient care via AI‐powered wearables. They highlighted AI's impact of AI through case studies and discussed challenges and solutions, ethical concerns, and the importance of data privacy. Furthermore, AI has facilitated advancements in precision medicine, a paradigm that tailors' medical treatments.^16^ Using AI algorithms to analyze genomic data, molecular profiles, and clinical phenotypes, researchers can identify disease biomarkers, predict treatment responses, and stratify patient populations for targeted therapies.^17^

AI‐powered predictive modeling techniques enable extracting meaningful insights from complex biomedical data, paving the way for personalized healthcare interventions and precision therapeutics.^18^ Moreover, AI has the potential to revolutionize healthcare delivery and patient engagement through digital health technologies and remote monitoring solutions. AI‐driven virtual health assistants, chatbots, and telemedicine platforms enable patients to access healthcare services remotely, receive medical advice, and manage chronic conditions. In addition, AI‐powered wearable devices and mobile health apps can monitor vital signs, track health metrics, and empower individuals to take proactive steps toward improving their health and wellness. From medical imaging interpretation and clinical decision support to precision medicine and digital health solutions, AI presents significant promise for advancing the goal of personalized, patient‐centered care.

The clinical need for AI in CIs is increasingly evident as healthcare providers seek to enhance the auditory experiences of individuals with hearing loss. CIs have revolutionized the treatment of severe to profound hearing loss, yet users often face significant challenges that can hinder their overall satisfaction and quality of life. One of the most pressing issues is poor speech perception in noisy environments, a common scenario in everyday life. Many CI users struggle to distinguish speech from background noise, leading to frustration, social withdrawal, and reduced communication effectiveness. While effective in quiet settings, traditional signal processing strategies in CIs often fall short in complex auditory environments, limiting the device's overall utility. In addition to improving speech perception, AI can also address issues related to device longevity and maintenance. Predictive maintenance algorithms can monitor the performance of CIs, identifying potential issues before they escalate into significant problems. This proactive approach minimizes device downtime and ensures that users can rely on their implants for consistent auditory support. Overall, integrating AI into CI technology represents a significant advancement in addressing the clinical challenges users face. By enhancing speech perception in noise, personalizing auditory experiences, and improving device reliability, AI can transform the landscape of aural rehabilitation, ultimately leading to better clinical outcomes and improved quality of life for individuals with hearing loss.^3^, ^19^, ^20^

The novelty of this paper lies in its comprehensive exploration of the intersection between CI technology and AI, presenting a transformative paradigm shift in auditory prosthetics. Unlike previous studies that primarily focused on the mechanical and acoustic aspects of CIs, this review emphasizes the integration of advanced AI algorithms and data‐driven approaches to enhance auditory outcomes. It highlights innovative developments such as noise reduction techniques, adaptive signal processing, and cognitive computing capabilities that empower users to manage their auditory health more effectively. Furthermore, the paper discusses the potential for personalized and intelligent devices that can adapt to individual user needs and environments, thereby improving speech perception and overall quality of life for CI users. This work underscores the importance of interdisciplinary collaboration among researchers, clinicians, and engineers by synthesizing current research and projecting future trends. It sets the stage for future innovations that could redefine auditory rehabilitation.

## 2 WORKFLOW

**FIGURE 2:**
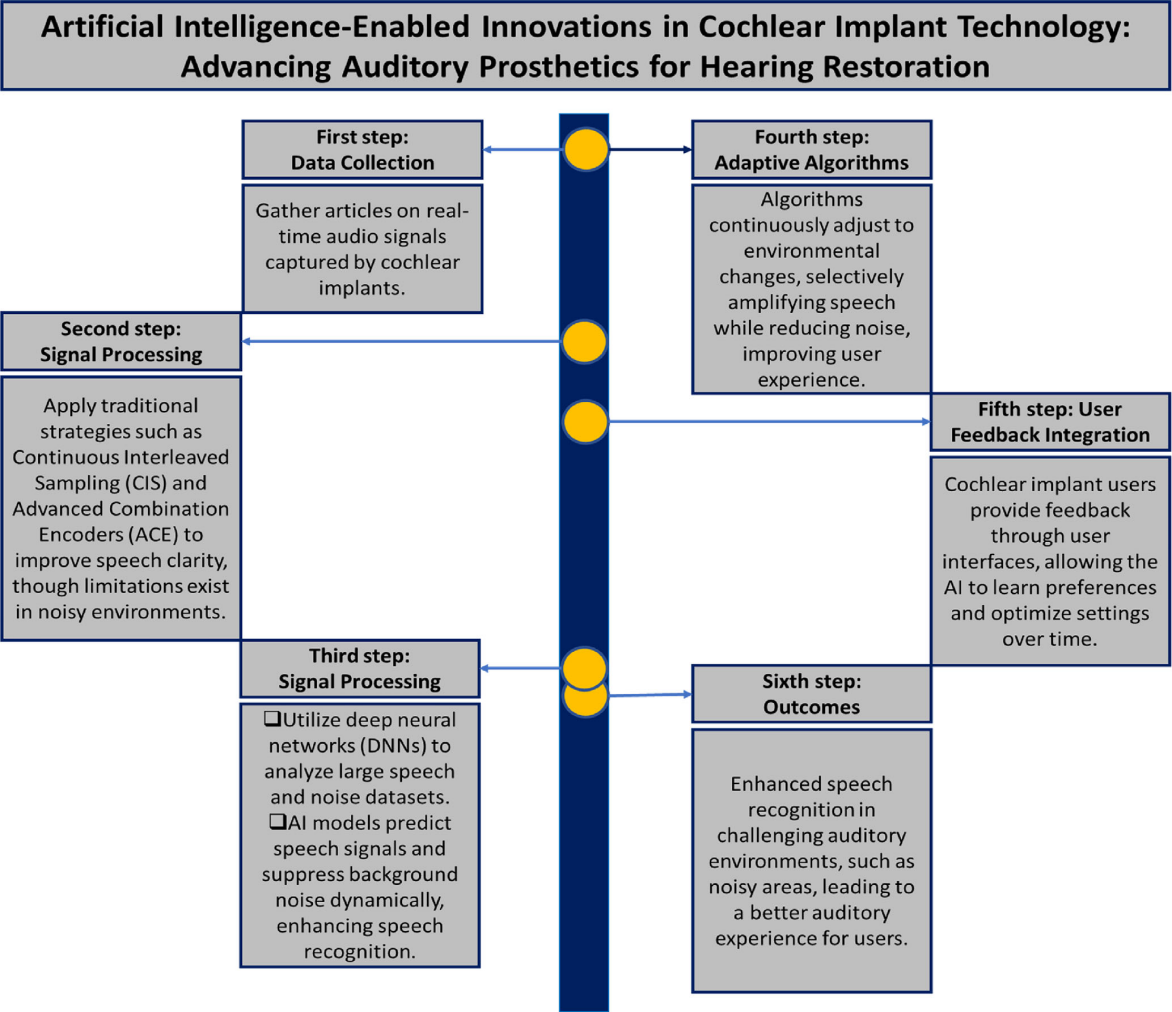
Workflow overview of AI‐driven innovations in cochlear implant technology: Enhancing hearing restoration through advanced signal processing and adaptive feedback mechanisms.

Advances in AI‐driven innovations are revolutionizing CI technology, paving the way for new solutions to restore hearing. The workflow presented in Figure 2 illustrates how this transformation unfolds. It begins with comprehensive data collection from real‐time audio signals captured by CIs. Traditional strategies, such as continuous interleaved sampling (CIS) and advanced combination encoders (ACE), are then applied to improve speech clarity during signal processing. While effective, these methods still face limitations in noisy environments.

AI‐based enhancements address these challenges by employing deep neural networks (DNNs) to analyze large datasets of speech and noise, thereby enabling dynamic speech signal prediction and background noise suppression. Adaptive algorithms play a pivotal role in this process by continuously adjusting to changing environmental conditions, and amplifying speech while reducing noise to enhance the overall user experience. Moreover, user feedback is integrated into the process, allowing CI users to provide input through user interfaces. This feedback enables the AI to learn individual preferences and refine settings over time.

The result of this AI‐driven approach is a marked improvement in speech recognition within complex auditory environments, leading to an enhanced auditory experience for users, particularly in noisy surroundings. This transformative shift in CI technology is not only improving the quality of life for individuals with hearing impairments but also expanding possibilities for future innovations in auditory prosthetics.

## 3 UNDERSTANDING HEARING: ANATOMY AND PHYSIOLOGY

**FIGURE 3:**
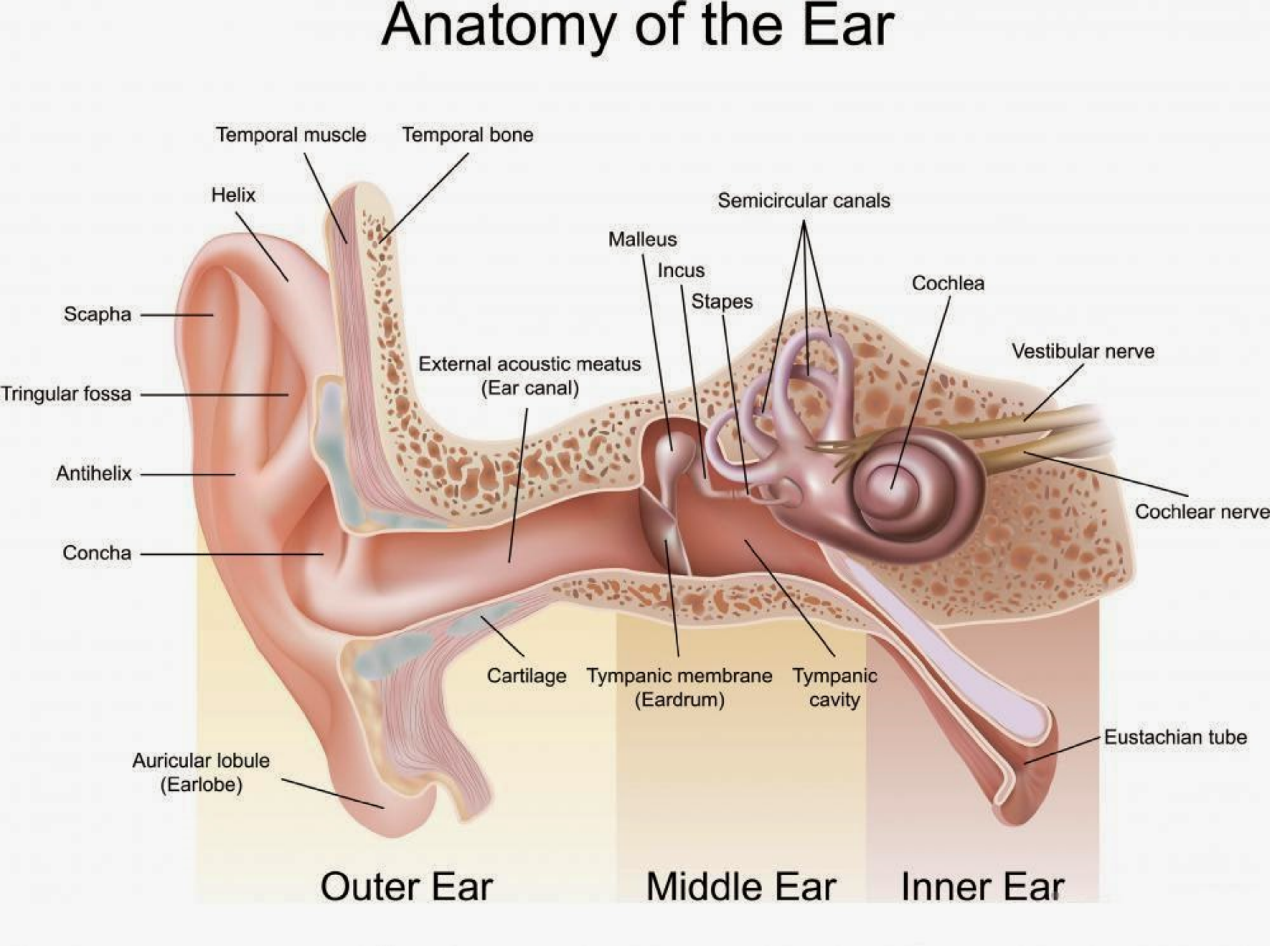
Anatomy of the human ear (copied from https://simplebiologyy.blogspot.com).

The human ear is a specialized organ that detects and interprets sounds and helps maintain balance. The ear is composed of three primary sections: the outer ear, middle ear, and inner ear, all of which are essential for transforming sound waves into electrical signals for processing by the brain. The outer ear comprises the pinna (auricle) and the external auditory canal (ear canal). The pinna, the visible part of the ear, collects sound waves and channels them into the ear canal. Its shape and folds assist in determining the direction of sound and aid sound localization. The external auditory canal is a tube‐like structure that generates sound waves from the pinna into the tympanic membrane (eardrum). This canal serves as a pathway for sound and protects the delicate structures inside the ear by producing earwax (cerumen), which traps dust and other particles. The ear has three primary parts, the outer, middle, and inner ear, which are crucial for converting sound waves into electrical signals for the brain (Figure 3).

The middle ear, an air‐filled space behind the eardrum, houses three tiny bones called ossicles: the malleus, incus, and stapes. The bones amplify and transmit sound vibrations from the eardrum to the inner ear. The eardrum vibrates in response to sound waves and these vibrations move through the malleus, incus, and stapes to the oval window of the inner ear. The Eustachian tube connects the middle ear to the nasopharynx, balancing the air pressure on both sides of the eardrum. The inner ear, a fluid‐filled area within the bony labyrinth, includes the cochlea, vestibule, and semicircular canals, which are essential for hearing and balance. The cochlea, resembling a snail's shell, has three fluid‐filled chambers: the scala vestibuli, scala media, and scala tympani. The organ of Corti in the scala media converts the mechanical vibrations into electrical signals. The movement of the stapes at the oval window creates pressure waves in the cochlear fluid, stimulating hair cells to send impulses to the auditory nerves and brain. The vestibular and semicircular canals manage balance and spatial orientation. The vestibule contains the utricle and the saccule, which detect linear acceleration and head position, whereas the semicircular canals detect rotational movement. The fluid and sensory hair cells in these canals sense changes in movement and send signals to the brain to maintain balance. Together, these structures process sound waves from the outer ear, amplify them in the middle ear, and convert them into electrical signals in the inner ear for the brain to interpret.^21^, ^22^, ^23^, ^24^, ^25^ Understanding these processes is crucial for developing auditory prosthetics and incorporating AI to enhance these technologies.

Hearing loss can be broadly categorized into conductive, sensorineural, and mixed.^26^ Conductive hearing loss occurs due to obstructions or damage to the outer or middle ear, which impedes the efficient transmission of sound waves to the inner ear. Common causes include earwax build‐up, ear infections (otitis media), perforated eardrum, otosclerosis (abnormal bone growth in the middle ear), and congenital malformations of the ear structures. These conditions lead to mechanical disruptions in sound transmission, such as fluid accumulation in the middle ear or fixation of the stapes bone, resulting in a reduction in the sound level and difficulty in hearing faint sounds. Treatment typically involves medical management, surgical intervention, or the use of hearing aids to amplify the sounds. However, sensorineural hearing loss arises from damage to the inner ear (cochlea) or the auditory nerve pathways to the brain and is often permanent. This type can be caused by genetic factors, aging (presbycusis), prolonged noise exposure, ototoxic medications, infections, and diseases, such as Ménière's disease and acoustic neuroma. This damage affects hair cells in the cochlea or auditory nerve fibers, leading to mild‐to‐significant hearing loss, with high‐frequency sounds often affected first. Management may include hearing aids, CIs, and medication or surgery for underlying conditions. Mixed hearing loss combines conductive and sensorineural loss elements, involving damage to both the outer/middle ear and inner ear/auditory nerve. For example, it can occur when an ear infection or earwax blockage complicates preexisting sensorineural hearing loss. Treatment requires addressing conductive and sensorineural components through medical, surgical, and rehabilitative approaches.^26^, ^27^, ^28^, ^29^, ^30^, ^31^, ^32^ Detailed information is provided in Table 1.

**TABLE 1:** Types of hearing loss and their management: A comprehensive summary.

Type of hearing loss	Causes	Pathophysiology	Effects	Treatment options
Conductive hearing Loss	Earwax buildup Ear infections (otitis media) Perforated eardrum Otosclerosis Congenital malformations	Mechanical disruptions impairing sound transmission Otitis media: Fluid accumulation dampens ossicles and tympanic membrane Otosclerosis: fixation of the stapes reduces sound transmission	Reduction in sound level Difficulty hearing faint sounds	Medical management Surgical intervention Hearing aids
Sensorineural hearing loss	Genetic factors Aging (presbycusis Noise exposure Ototoxic medications Infections Ménière's disease Acoustic neuroma	Damage to hair cells in the cochlea or auditory nerve fibers Noise: Hair cell damage reduces ability to convert vibrations to electrical signals Ototoxic substances: Cellular damage in cochlea Genetic mutations: Affect development/function of cochlea and auditory pathways Infections/autoimmune conditions: Inflammation and damage to inner ear structures	Mild to significant hearing loss Typically permanent Often affects high‐frequency sounds first	Hearing aids CIs Medication or surgery for underlying conditions
Mixed hearing loss	Combination of conductive and sensorineural causes (e.g., ear infection or earwax blockage in a person with sensorineural hearing loss)	Damage in both outer/middle ear and inner ear/auditory nerve	Combination of conductive and sensorineural effects	Combination of treatments for both conductive and sensorineural component Medical, surgical, and rehabilitative approaches

**FIGURE 4:**
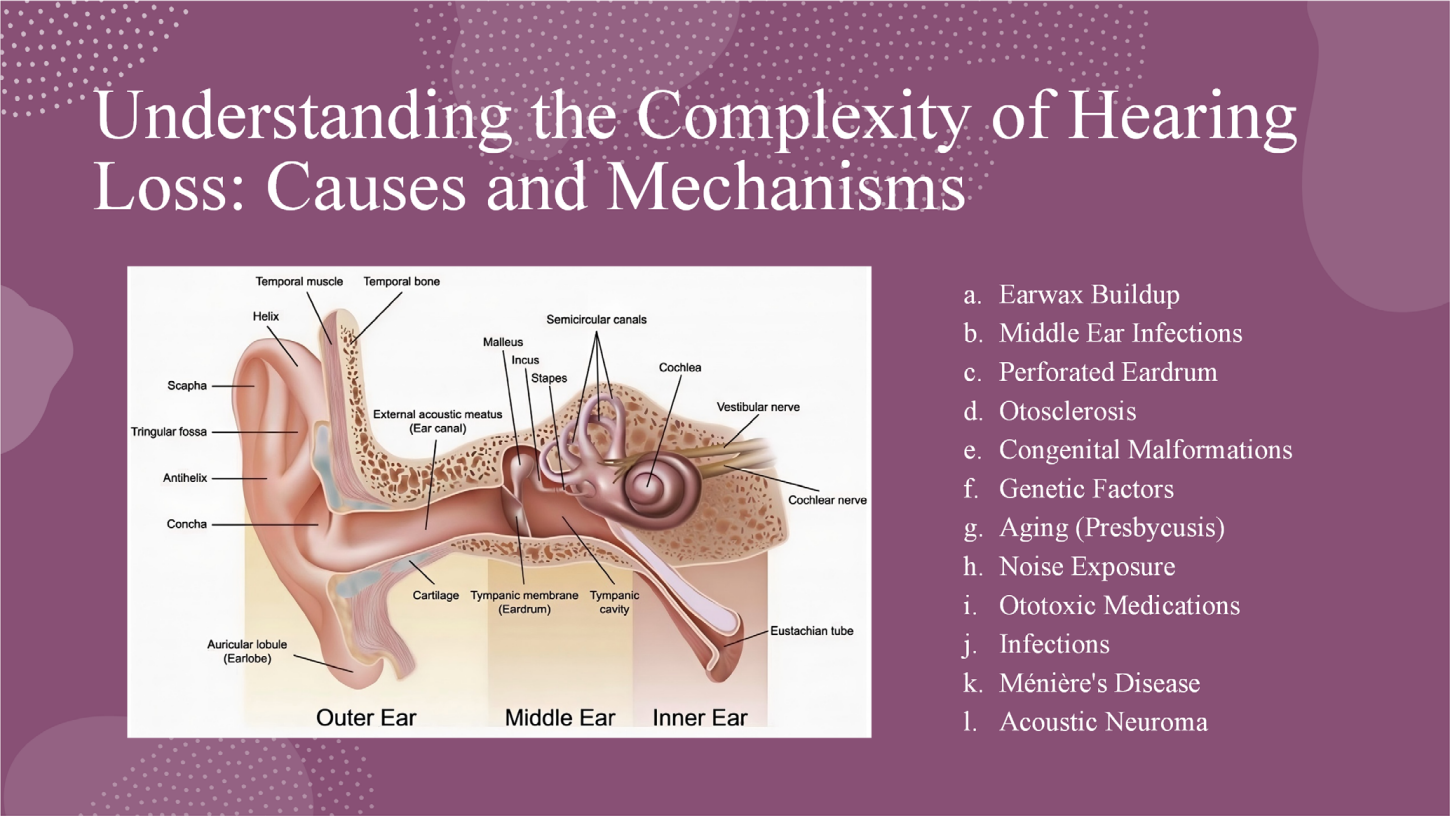
The complex causes of hearing loss and their mechanisms (the idea is captured from Wang and Puel^36^ and Sohmer^37^ and https://onthebaymagazine.com/see‐hear/).

Hearing loss can be attributed to various causes, each with distinct pathophysiological mechanisms. Earwax buildup or cerumen accumulation in the ear canal blocks sound transmission, creating a mechanical obstruction that impairs hearing. Ear infections, which are particularly common in children, lead to fluid buildup and inflammation in the middle ear, which dampens the movement of the ossicles (tiny bones in the ear) and the eardrum, thereby reducing sound conduction. A perforated eardrum, characterized by a hole or tear, directly disrupts the normal transmission of sound waves. Otosclerosis, which involves abnormal bone growth around the stapes bone in the middle ear, restricts the movement of the bone and diminishes its ability to transmit sound vibrations to the cochlea. Congenital malformations of ear structures present during birth also cause mechanical disruptions in sound transmission. Genetic factors can lead to congenital hearing loss at birth or to progressive hearing loss that develops later in life. Aging (presbycusis) causes a gradual decline in hearing ability, typically starting with high‐frequency sounds, due to the age‐related degeneration of hair cells in the cochlea. Prolonged exposure to loud noise damages these hair cells, reducing their ability to convert mechanical sound vibrations into electrical signals and leading to noise‐induced hearing loss. Ototoxic medications such as certain antibiotics and chemotherapeutic drugs cause cellular damage to the cochlea and impair hearing. Infections, such as meningitis, can result in inflammation and permanent damage to the cochlea, leading to hearing loss. Ménière's involves abnormal fluid buildup in the inner ear, causing fluctuating hearing loss, tinnitus (ringing in the ears), and vertigo (a spinning sensation). An acoustic neuroma, a benign tumor of the auditory nerve, compresses the nerve and disrupts hearing (Figure 4). These causes and their underlying mechanisms highlight the diverse and complex nature of hearing loss.^33^, ^34^, ^35^

## 4 EVOLUTION OF CI TECHNOLOGY

CIs have revolutionized the management of severe to significant hearing loss by providing auditory perception to individuals who receive limited or no benefit from conventional hearing aids. The development of CIs technology has undergone significant advancements since its inception, driven by continuous research and technological innovations.^38^, ^39^, ^40^ The evolution of CIs can be categorized into several key phases: Early experimental devices, the advent of commercial implants, improvements in speech‐processing strategies, and recent advancements in AI and wireless technologies. The concept of electrical stimulation of the auditory nerve to produce hearing sensations dates back to the 18th century.^41^ However, the first documented attempt to develop a functional CI was made in the 1950s. André Djourno and Charles Eyriès in France experimented with electrical stimulation of the auditory nerve, laying the foundation for future developments. Although the early devices were rudimentary, they demonstrated the feasibility of electrically induced hearing. These initial efforts faced skepticism and ethical concerns, as many believed electrical stimulation could not provide meaningful hearing. Although this offered essential sound awareness and improved lip‐reading abilities, it was limited to conveying complex speech information. Researchers have developed multichannel implants that provide more detailed auditory information by stimulating different parts of the cochlea. Multichannel implants have marked significant advancements, enabling users to perceive speech with improved clarity and understanding in quiet environments.^42^, ^43^, ^44^

**FIGURE 5:**
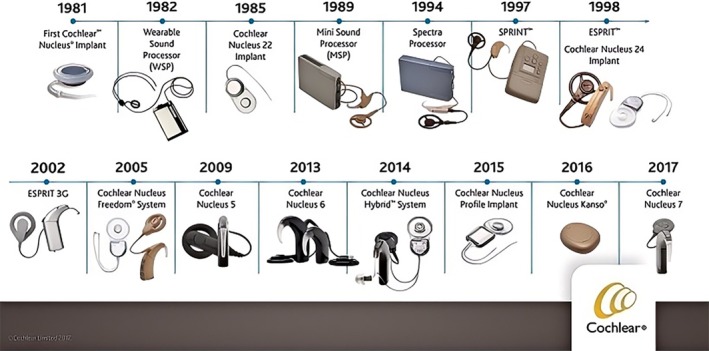
History of CIs over the previous four decades (copied from https://earcommunity.org).

CIS and ACE improve speech perception by effectively encoding sound information. A CIS uses the rapid, sequential stimulation of electrode contacts, minimizes channel interactions, and enhances the temporal resolution of speech signals. ACE combines the CIS features with higher stimulation rates to provide users with improved speech recognition, especially in noisy environments. These developments significantly enhance the performance of CIs, making them more practical for a broad range of users.^45^, ^46^ The history of CIs over the last four decades is shown in Figure 5.

Recent advancements in CI technology have focused on integrating AI and wireless capabilities. AI‐driven algorithms have been developed to improve sound processing, noise reduction, and speech recognition. ML techniques allow implants to adapt to individual user preferences and listening environments, thereby providing a more personalized auditory experience.^47^, ^48^, ^49^, ^50^ Wireless technology has also transformed CIs, allowing direct streaming from smartphones, televisions, and other devices. This connectivity enhances the usability and convenience of CIs, seamlessly integrating them into users' daily lives. In addition, wireless capabilities facilitate remote programming and monitoring, enabling audiologists to make adjustments and provide support without requiring in‐person visits.^51^ Incorporating AI and wireless technologies represents a significant advancement in CI development. These innovations promise to enhance the quality of life of individuals with hearing loss by offering improved sound quality, significant convenience, and personalized auditory experiences. In conclusion, the evolution of CI technology reflects decades of relentless research and innovation. From early experimental devices to sophisticated multichannel implants and the integration of AI and wireless capabilities, CIs have continuously advanced to provide better auditory outcomes for users. These developments highlight the remarkable progress made in the field of auditory prosthetics and underscore the potential for future breakthroughs in enhancing hearing health.^52^

### 4.1 Historical perspective

The history of hearing loss treatments and the development of CIs shows persistent innovation and notable dedication to improving the quality of life of those with hearing impairments. This journey began long before the modern era of sophisticated medical devices, with early attempts to understand and manage hearing loss through rudimentary means, and evolved into the advanced, highly effective CI technologies we have today. The understanding of hearing and hearing loss dates back to ancient civilizations. Early attempts to treat hearing loss included herbal remedies and rudimentary ear surgeries. However, these early methods were often ineffective and lacked a scientific basis.^46^ The invention of the ear trumpet in the 17th century was a significant milestone. This simple device, essentially a funnel that amplifies sound, provides the first real solution for individuals with hearing loss, albeit limited. The 19th century saw further advancements in the development of more sophisticated hearing aids. The first electronic hearing aids emerged following the invention of the telephone and microphone by Alexander Graham Bell and Thomas Edison.^53^ These electronic devices, cumbersome and limited in amplification and frequency ranges, represented a significant leap forward from purely mechanical devices. The mid‐20th century was a pivotal period for auditory research. The idea of directly stimulating the auditory nerve to restore hearing has begun to take shape. In the 1950s, André Djourno and Charles Eyriès conducted pioneering work in France using electrical stimulation to elicit auditory sensations in deaf individuals. Their experiments demonstrated that it was possible to bypass the damaged parts of the ear and directly stimulate the auditory nerve, paving the way for future developments.^54^

In recent years, the integration of AI and wireless technology has driven the evolution of CIs to new heights. AI algorithms have been developed to enhance sound processing, noise reduction, and speech recognition.^55^, ^56^ ML techniques enable implants to adapt to individual user preferences and changing acoustic environments, thereby providing a more personalized auditory experience.^57^ Wireless technology has also transformed CIs, facilitating direct streaming through smartphones, televisions, and other devices. This connectivity enhances the convenience and usability of CIs, making them more integrated into users' everyday lives. In addition, remote programming and monitoring capabilities have been developed, allowing audiologists to adjust implant settings and provide support without requiring in‐person visits.^58^ The historical perspective of CIs is a testament to the relentless pursuit of innovation in auditory prosthetics. From early experimental devices to advanced multichannel implants and the integration of cutting‐edge AI and wireless technologies, CIs have evolved continually to provide better auditory outcomes for individuals with hearing loss.

### 4.2 Components and working principles

CIs are intricate medical devices designed to provide a sense of sound to individuals with severe to significant sensorineural hearing loss. Unlike traditional hearing aids, which amplify sound, CIs bypass damaged parts of the ear and directly stimulate the auditory nerves. Understanding the components and working principles of CIs is essential for understanding how these devices can restore hearing. This section explores the primary components of CIs and their operational mechanisms.^59^

#### 4.2.1 External components

Table 2 lists the external components of CIs, detailing their functions and descriptions.^60^

**TABLE 2:** Overview of external components in CIs.

External component	Function	Description
Microphone	Captures sound from the environment and converts it into electrical signals	Typically housed in a behind‐the‐ear unit, similar to a hearing aid
Speech processor	Receives electrical signals from the microphone, filters and processes them to extract relevant speech and environmental sounds	Located in the external unit, applies advanced algorithms to enhance speech clarity and reduce background noise
Transmitter coil	Sends processed signals to the internal components of the cochlear implant via radio frequency (RF) waves	Worn on the scalp, held in place by a magnet. Sends the processed sound signals to the internal components of the cochlear implant for further processing

#### 4.2.2 Internal components

The internal components of a CI include a receiver/stimulator and an electrode array (Table 3).^61^

**TABLE 3:** Overview of internal components in CIs.

Internal component	Function	Description
Receiver/stimulator	Surgically implanted under the skin, just behind the ear. Receives RF signals from the external transmitter coil and converts them into electrical impulses	Designed to interface with the body safely and effectively, ensuring long‐term functionality
Electrode array	Surgically inserted into the cochlea, the spiral‐shaped organ in the inner ear. Contains multiple electrodes that extend along the length of the cochlea	Flexible, thin wire that delivers electrical impulses to specific locations within the cochlea, corresponding to different frequencies of sound

#### 4.2.3 Working principles

A CI operation involves several steps, from sound capture to auditory nerve stimulation. Here, we provide a detailed look at the working principles:

1. *Sound capture and processing*: The process begins when a microphone captures sound waves and converts them into electrical signals. These signals are sent to a speech processor that uses digital signal‐processing algorithms to analyze and filter sounds. The processor extracts important speech features, such as pitch, intensity, and timing, while minimizing background noise.^62^
2. *Transmission to Internal Components*: The processed signals were transmitted from the speech processor to the transmitter coil. The coil sends these signals as RF waves through the skin to the implanted receiver or stimulator. The skin has minimal impedance to RF waves, which allows efficient signal transmission.^63^
3. *Conversion to electrical impulses*: The receiver or stimulator receives RF signals and converts them into coded electrical impulses. These impulses are then sent to an electrode array implanted in the cochlea. Each electrode corresponded to a specific frequency range that mimicked the tonotopic organization of the natural cochlea.^64^
4. *Stimulation of the auditory nerve*: The electrodes in the array delivered precise electrical impulses to the auditory nerve fibers within the cochlea. This electrical stimulation bypasses damaged or nonfunctional hair cells and directly activates the auditory nerve. The brain interprets these electrical signals as sound, allowing the user to perceive speech and environmental noises.^65^

#### 4.2.4 Signal processing strategies

Signal‐processing strategies are crucial in determining the effectiveness of a CI. Several techniques have been developed to optimize sound quality and speech comprehension. CIS is a widely used strategy for rapidly and sequentially delivering electrical pulses to different electrodes. This reduces the channel interactions and enhances the temporal resolution of the perceived sound. ACEs combine the features of CIS with high stimulation rates. It selects a sound signal's most salient frequency components and delivers them to the corresponding electrodes, thereby improving speech recognition, particularly in noisy environments. High‐resolution (HiRes) strategy provides a higher number of stimulation cycles per second, offering significant details of electrical stimulation patterns. This can improve sound quality and speech comprehension.^50^, ^66^

The future of CI technology lies in the integration of advanced computational algorithms and AI. AI can enhance the adaptability of CIs, allowing them to adjust to varying acoustic environments and user preferences automatically. In addition, wireless connectivity features enable seamless streaming from personal electronic devices, further improving user experience. In conclusion, CIs are complex devices that restore hearing by directly stimulating the auditory nerve using electrical impulses. The coordinated function of the external and internal components, along with sophisticated signal processing strategies, allows CIs to provide meaningful auditory perception to individuals with severe hearing loss. Ongoing technological advancements promise to enhance the capabilities and outcomes of CIs further, offering new hope for those with hearing impairments.

### 4.3 Contemporary challenges and limitations

Despite remarkable advancements in CI technology, several challenges and limitations persist. These challenges include technical constraints on biological factors that affect the overall efficacy and accessibility of CIs for users. Addressing these issues is crucial for improving outcomes and expanding the benefits of CIs to a broader population. Table 4 summarizes the challenges and limitations of CI technology, highlighting the diverse issues affecting its efficacy and accessibility for users.

**TABLE 4:** Overview of contemporary challenges and limitations in cochlear implant technology.

Challenges and limitations	Description
Variability in patient outcomes	Outcome variability influenced by factors such as duration and degree of hearing loss, age at implantation, and condition of auditory nerve and cochlea. Early implantation generally yields better results, but outcomes can be unpredictable.^67^
Speech perception in noise	Difficulty understanding speech in noisy environments despite advancements in signal processing strategies. Ongoing research aims to improve noise reduction techniques and enhance speech recognition in challenging acoustic conditions.^68^
Preservation of Residual Hearing	Concerns about potential damage to residual hearing during cochlear implantation. Advances in surgical techniques and electrode array design aim to minimize this risk, but consistent preservation of residual hearing remains a challenge.^69^
Device longevity and reliability	Ensuring the long‐term reliability and durability of CIs, including material degradation and component failure. Regular technological advancements can make older implants obsolete, posing challenges for users requiring upgrades.^70^
Access and affordability	Limited access to CIs and associated services, particularly in low‐ and middle‐income countries, due to high costs, lack of infrastructure, and trained professionals. Efforts to reduce costs and expand training programs are ongoing.^71^
Ethical and social considerations	Ethical dilemmas surrounding the use of CIs, particularly regarding the Deaf community's cultural identity and decision‐making processes for implanting young children. Respecting diverse perspectives and promoting inclusive decision‐making is crucial.^72^
Technological integration and user experience	Challenges in integrating new features and ensuring a positive user experience with modern CIs, particularly for older adults or those less familiar with digital technologies. User‐friendly designs and comprehensive support are essential.^73^

Despite advancements in CI technology, several challenges that impact user experience and clinical outcomes persist. One major issue is speech perception in noisy environments. AI‐driven noise reduction algorithms can enhance sound processing by effectively differentiating between speech and background noise, improving communication abilities and social engagement. Device longevity is another concern; predictive maintenance powered by AI can monitor performance and usage patterns, allowing for timely interventions that minimize downtime and enhance reliability. Personalization of the auditory experience is crucial, as AI can facilitate adaptive mapping strategies that continuously learn from user feedback, leading to improved satisfaction and speech perception. Additionally, AI can enhance connectivity with smart devices, ensuring seamless integration and better usability in daily life. Lastly, cognitive load and auditory fatigue can be mitigated through cognitive hearing assistants that filter irrelevant sounds and provide contextual information, reducing cognitive overload and enhancing listening comfort. Overall, these AI applications offer significant potential to address contemporary challenges in CI technology, ultimately improving the quality of life for users.

## 5 AI APPLICATIONS IN AUDITORY PROSTHETICS

Integrating AI into auditory prosthetics, particularly CIs, represents a significant advancement in hearing restoration.^74^, ^75^ AI technologies can enhance CI's performance, adaptability, and user experience. One primary application of AI in CIs is the enhancement of sound processing algorithms. Traditional strategies, such as CIS and ACEs, have limitations in noisy environments; however, AI‐based algorithms can significantly improve speech recognition and sound quality by learning to differentiate between speech and background noise more effectively.^49^, ^75^ These AI algorithms use deep‐learning techniques to analyze large datasets of speech and noise, thereby enabling the development of models that can predict and enhance speech signals. This adaptive approach helps users better understand speech in challenging listening situations such as crowded places or noisy streets. In addition, AI facilitates the customization of CIs to meet individual user needs and preferences. By continuously monitoring auditory input and user interactions, AI systems can learn and adapt to the specific hearing preferences of each user, thereby enhancing user satisfaction and the overall hearing experience. Another significant application of AI in CIs is the improvement of noise‐reduction algorithms. Background noise remains a challenge for users; however, AI‐powered noise reduction algorithms offer a promising solution by providing more sophisticated and effective noise management. These algorithms use ML models trained on extensive datasets to identify and suppress various types of noise while preserving speech signals. Moreover, AI enables real‐time sound classification and enhances the situational awareness of CI users by identifying and categorizing different types of sounds. Sound classification systems use deep‐learning models to analyze incoming sounds and provide users with valuable information about their surroundings. AI‐driven remote monitoring and adjustment capabilities offer significant benefits for CI users and healthcare providers. Remote monitoring systems track and analyze the performance of implants, allowing healthcare providers to make informed decisions regarding adjustments and interventions. AI can also facilitate remote adjustment of implant settings, reducing the need for frequent in‐person visits. In addition, AI plays a crucial role in predictive maintenance, ensuring the longevity and reliability of CIs by analyzing data from implant sensors and usage patterns to predict potential device failures or performance issues before they occur. Finally, AI‐powered rehabilitation and training tools can transform the post‐implantation process for CI users by providing personalized and effective rehabilitation programs tailored to the specific needs and progress of each user. These tools employ interactive and adaptive technologies to help users improve their auditory skills and adapt to implants, thereby enhancing their overall QoL.

### 5.1 Speech enhancement and noise reduction

The integration of advanced signal processing techniques is pivotal in CI technology to enhance speech clarity and mitigate background noise, thereby improving the overall auditory perception of users.^50^, ^76^ This section discusses the methodologies and technologies employed for speech enhancement and noise reduction in CIs (Table 5).

**TABLE 5:** Overview of speech enhancement and noise reduction techniques in CIs.

Methodology	Description
Signal processing algorithms	Algorithms analyze incoming audio signals to enhance speech clarity and reduce background noise. Traditional methods like CIS and ACEs are effective in tranquil environments but less so in noisy surroundings.^77^
Adaptive noise reduction	Algorithms dynamically adjust parameters based on ambient noise and user preferences, leveraging ML to differentiate speech from noise. By reducing background noise while preserving speech, they significantly improve speech intelligibility, enabling communication in challenging settings.^68^
Spectral Subtraction	This technique estimates background noise's spectral profile and subtracts it from the original signal to retain speech components. Effective for attenuating stationary noise, such as air conditioning hum or traffic noise, spectral subtraction offers real‐time implementation in CIs.^78^
Wiener filtering	Wiener filtering estimates clean speech from noisy signals using statistical models, exploiting the statistical correlation between speech and noise. Beneficial in environments with fluctuating or non‐stationary noise, Wiener filtering enhances speech intelligibility in CIs.^79^
Deep‐learning techniques	Advancements in deep learning, including deep neural networks (DNNs) and convolutional neural networks (CNNs), revolutionize speech enhancement and noise reduction. These techniques offer sophisticated methods to improve speech perception in CIs, marking a significant leap forward in overcoming challenges posed by background noise in various environments.^80^

### 5.2 Advanced signal processing techniques

Advanced signal‐processing techniques play a crucial role in optimizing the performance of CIs, particularly in challenging listening environments. These techniques use sophisticated algorithms and adaptive strategies to enhance speech perception and mitigate the effects of background noise. One prominent approach is the application of adaptive beamforming algorithms that selectively focus on the desired sound source while suppressing interfering noise sources.^50^ By dynamically adjusting the directional sensitivity of the microphone array, adaptive beamforming algorithms can improve speech intelligibility in noisy environments, such as crowded gatherings or bustling streets. Another key technique involves the integration of ML algorithms for personalized sound processing. These algorithms analyze individual listening preferences and environmental factors to optimize implant's settings in real time and provide users with a tailored auditory experience. Furthermore, the incorporation of binaural processing techniques enhances the spatial hearing and localization abilities for CI users. By simulating the natural binaural cues present in normal hearing, such as interaural time and level differences, binaural processing algorithms can improve the perception of sound direction and spatial awareness. In addition, the advent of deep‐learning approaches has revolutionized signal processing in CIs. DNNs trained on large speech and noise datasets can effectively extract meaningful features and adaptively enhance speech signals while suppressing background noise. These deep‐learning‐based algorithms demonstrate superior performance in complex listening scenarios, offering a promising avenue for further advancements in CI technology. Collectively, the integration of advanced signal processing techniques has immense potential for improving auditory outcomes and quality of life for CI users.

### 5.3 Personalized rehabilitation strategies

Personalized rehabilitation strategies play a pivotal role in optimizing CI outcomes of cochlear implantation by tailoring auditory training programs to the unique needs and abilities of individual users. These strategies encompass a range of interventions aimed at improving speech perception, auditory skills, and communication abilities. One key component of personalized rehabilitation is the incorporation of speech‐training exercises that target specific areas of difficulty for CI users, such as speech recognition in noisy environments or the perception of subtle phonetic contrasts. By systematically addressing these challenges through targeted exercises, users can gradually improve their speech comprehension and communication proficiency. Auditory training programs often include activities designed to enhance sound localization and spatial awareness, leveraging virtual reality or computer‐based simulations to create immersive auditory environments. These exercises help users develop a more accurate sense of sound direction and spatial relationships, which are crucial for navigating complex auditory scenes. Moreover, personalized rehabilitation strategies integrate psychosocial support and counseling to address the emotional and social aspects of hearing loss and cochlear implantation. By providing guidance and resources to cope with the psychosocial impact of hearing loss, rehabilitation professionals can empower users to navigate social interactions and build confidence in their communication abilities. Technological advances have enabled the development of mobile applications and remote monitoring tools that facilitate home‐based rehabilitation and allow users to track their progress over time. These personalized rehabilitation strategies, tailored to the individual needs and preferences of CI users, play a vital role in maximizing the benefits of cochlear implantation and enhancing the overall quality of life.

## 6 INTEGRATION OF AI WITH CIs

Integrating AI with CIs represents a promising frontier in auditory prosthetics, offering opportunities to enhance speech perception, optimize device performance, and personalize user experience. AI‐powered algorithms leverage ML and neural networks to analyze and process auditory signals in real‐time, enabling adaptive and context‐aware adjustments to CI settings. A key application of AI in CIs is the development of adaptive sound processing strategies that dynamically adjust signal processing parameters based on the user's auditory environment and listening preferences. By continuously monitoring and analyzing incoming auditory signals, AI algorithms can optimize speech processing and noise reduction to improve speech intelligibility and enhance user satisfaction.^81^ In addition, AI‐driven optimization techniques enable personalized mapping strategies that adapt to individual users' auditory needs and preferences. These personalized mappings consider factors such as electrode impedance, neural survival, and speech perception abilities, resulting in improved auditory outcomes for CI users. Furthermore, AI‐based predictive modeling facilitates the proactive maintenance and troubleshooting of CI devices, detecting and addressing potential issues before they affect the device performance. Remote monitoring systems powered by AI algorithms enable healthcare providers to remotely assess device functionality and user performance, facilitating timely interventions and adjustments. Moreover, AI‐enabled speech recognition technologies present promise for enhancing speech comprehension in challenging listening environments by improving the ability of CI users to process and interpret complex speech signals. By harnessing the power of AI, CIs can be transformed into intelligent adaptive devices that provide enhanced auditory experiences and an improved quality of life for users.^82^, ^83^

### 6.1 Real‐time adaptive control systems

Table 6 provides a concise overview of the components and functionalities of the real‐time adaptive control systems used in CI technology. Each component is briefly described, highlighting its role in optimizing the device performance and enhancing the user experience.

**TABLE 6:** Overview of components and functionalities in real‐time adaptive control systems for CIs.

Component	Description
Adaptive signal processing algorithms	Dynamically adjust parameters based on real‐time input and user feedback to optimize speech perception and reduce background noise effects. Adapt processing strategies to enhance user experience by analyzing incoming auditory signals.^84^
Neural network‐based adaptation	Apply artificial neural networks to learn and adapt to individual user preferences over time. Continuously update mapping strategies and processing parameters for personalized and optimized device performance, catering to unique auditory needs.^85^
Feedback mechanisms	Assess user satisfaction and device performance accurately through effective feedback mechanisms, including user‐reported feedback, sensor data, and physiological measures. Integrate multiple feedback sources to iteratively refine device settings and enhance overall performance.^86^
Dynamic parameter adjustment	Enable dynamic adjustment of device parameters in response to changes in the auditory environment. Automatically switch between processing strategies or adjust stimulation parameters to optimize speech perception in challenging listening situations.^87^
Continuous improvement and learning	Continuously learn and improve over time by analyzing user interactions and outcomes. Refine algorithms and adaptation strategies to better meet the needs of cochlear implant users, ensuring maximum benefit from the devices. Enhance device performance and optimize user experience in various listening environments through iterative improvement.^88^

### 6.2 Cognitive hearing assistants

Cognitive hearing assistants represent a novel approach to augment the capabilities of CIs by leveraging AI and ML algorithms to provide personalized and adaptive support to users in real‐time. These assistants integrate advanced signal‐processing techniques with cognitive computing capabilities to enhance speech perception, improve sound localization, and optimize the auditory experience for users. Table 7 offers a comprehensive understanding of the capabilities of cognitive hearing assistants that enhance the auditory experiences of CI users.

**TABLE 7:** Overview of functionalities in cognitive hearing assistants for CIs.

Functionality	Description
AI‐based speech enhancement	Utilizes deep‐learning‐based algorithms to analyze and enhance speech signals in real time, improving speech intelligibility and clarity for users. Selectively amplifies speech components while suppressing background noise to aid understanding in challenging listening environments.^89^
Context‐aware sound processing	Adaptively adjusts device settings based on the user's environment and listening preferences by analyzing contextual cues such as noise levels, speaker proximity, and user activity. Optimizes signal processing parameters to provide the best possible auditory experience in any situation.^90^
Personalized recommendations and feedback	Provides personalized recommendations and feedback to users by learning from user interactions and preferences. Tailors' recommendations for device settings, auditory training exercises, and communication strategies. Empowers users to make informed decisions about their auditory health by offering real‐time feedback on speech perception and device usage.^91^
Integration with wearable devices	Integrates with wearable devices such as smartphones, smartwatches, and hearing aid accessories to enhance functionality and accessibility. Users can adjust device settings, receive notifications, and access personalized auditory training programs directly from their wearable devices.^92^
Continuous learning and improvement	Continuously learns and improves over time through ML algorithms. Analyzes user feedback, device performance data, and environmental factors to refine algorithms and better meet the evolving needs of users. Ensures users receive the most effective support and assistance from their cognitive hearing assistants.^88^

## 7 CONCLUSION

This review highlights the transformative potential of integrating CIs with AI and data‐driven approaches in revolutionizing auditory prosthetics. By examining the structure and function of the human ear, the mechanisms of hearing loss, and the evolution of CI technology, we underscore the profound implications of this convergence for clinical practice and user experience.

Integrating AI enhances personalized mapping strategies and adaptive signal processing, significantly improving speech perception and user satisfaction. Predictive maintenance techniques facilitate proactive management of device issues, minimizing downtime and optimizing performance. Continuous monitoring of user outcomes and electronic health records empowers clinicians to make evidence‐based decisions tailored to individual needs.

Ultimately, the convergence of CIs, AI, and data‐driven approaches represents a paradigm shift in auditory rehabilitation, offering users a more personalized, adaptive, and intelligent auditory experience. This innovation enhances auditory outcomes and significantly improves the quality of life for individuals with hearing loss, enabling them to navigate diverse listening environments with greater ease and confidence. As research and collaboration in this field continue to advance, we are optimistic about the future of auditory health and the potential for further breakthroughs that will enrich the lives of CI users worldwide.

## AUTHOR CONTRIBUTIONS

**Guodao Zhang:** Conceptualization; writing – original draft; writing – review and editing; validation; visualization; investigation; formal analysis; software; resources; methodology; funding acquisition; project administration; data curation. **Rui Chen:** Conceptualization; investigation; visualization; writing – original draft; writing – review and editing; software; formal analysis; project administration; data curation; resources; methodology; validation. **Hamzeh Ghorbani:** Conceptualization; investigation; writing – original draft; writing – review and editing; visualization; validation; methodology; supervision; resources; data curation; formal analysis; project administration; software. **Wanqing Li:** Writing – original draft; writing – review and editing; project administration; formal analysis; data curation; resources; software; methodology; validation; investigation; conceptualization; funding acquisition. **Arsen Minasyan:** Writing – original draft; writing – review and editing; visualization; software; supervision; resources; investigation; conceptualization; validation; methodology; funding acquisition. **Yideng Huang:** Writing – original draft; writing – review and editing; project administration; resources; data curation; software; methodology; validation; visualization; investigation. **Sen Lin:** Writing – original draft; writing – review and editing; visualization; formal analysis; software; methodology; conceptualization; data curation; resources. **Minmin Shao:** Writing – original draft; writing – review and editing; visualization; validation; formal analysis; software; conceptualization; data curation.

## CONFLICT OF INTEREST STATEMENT

The authors declare no conflicts of interest regarding the publication of this article.

## Data Availability

The data that support the findings of this study are available from the corresponding author upon reasonable request.
